# The relationship between pancreatic cancer and hypercoagulability: a comprehensive review on epidemiological and biological issues

**DOI:** 10.1038/s41416-019-0510-x

**Published:** 2019-07-22

**Authors:** Elena Campello, Anton Ilich, Paolo Simioni, Nigel S. Key

**Affiliations:** 1 Thrombotic and Haemorrhagic Disease Unit, Department of Medicine, University of Padova, Padova, Italy; 2 Division of Haematology/Oncology, Department of Medicine, University of North Carolina at Chapel Hill, Chapel Hill, NC USA

**Keywords:** Tumour biomarkers, Risk factors, Outcomes research

## Abstract

It has long been recognised that pancreatic cancer induces a hypercoagulable state that may lead to clinically apparent thrombosis. Although the relationship between pancreatic cancer and hypercoagulability is well described, the underlying pathological mechanism(s) and the interplay between these pathways remain a matter of intensive study. This review summarises existing data on epidemiology and pathogenesis of thrombotic complications in pancreatic cancer with a particular emphasis on novel pathophysiological pathways. Pancreatic cancer is characterised by high tumoural expression of tissue factor, activation of leukocytes with the release of neutrophil extracellular traps, the dissemination of tumour-derived microvesicles that promote hypercoagulability and increased platelet activation. Furthermore, other coagulation pathways probably contribute to these processes, such as those that involve heparanase, podoplanin and hypofibrinolysis. In the era in which heparin and its derivatives—the currently recommended therapy for cancer-associated thrombosis—might be superseded by direct oral anticoagulants, novel data from mouse models of cancer-associated thrombosis suggest the possibility of future personalised therapeutic approaches. In this dynamic era for cancer-associated thrombosis, the discovery of novel prothrombotic and proinflammatory mechanisms will potentially uncover pharmacological targets to prevent and treat thrombosis without adversely affecting haemostasis.

## Background

Pancreatic cancer is the seventh most common cause of cancer-related death worldwide,^1^ with a standardised global incidence rate of 4.2 cases per 100,000 inhabitants. The incidence rates of pancreatic cancer are relatively higher in North America (7.4/100,000) and Western Europe (7.3/100,000), followed by the rest of Europe, Australia and New Zealand (about 6.5/100,000), whereas the lowest rates are observed in Africa, the Middle East and Central Asia (<1.5/100,000),^1^ possibly due to genetic, somatometric and dietary differences.

In 1865, the French physician, Armand Trousseau, who ultimately diagnosed his own gastric malignancy after developing thrombophlebitis in his upper extremity, described that migratory venous thromboses may complicate the course of malignancies.^2^ It has since been established that pancreatic cancer has a peculiar and unique ability to induce a hypercoagulable state that is associated with clinically significant thrombosis in patients—that is, their blood has an abnormal tendency to coagulate, thereby conferring an increased risk of developing clots. A number of risk factors for venous and arterial thrombosis have been identified in cancer patients. These include certain comorbidities, surgery, immobility, tumour histology and stage, the presence of indwelling central venous catheters, and chemotherapy and/or some molecular targeted therapies.^3^ These risk factors, which are usually classified as patient-, tumour- or treatment-related, cumulatively induce a systemic hypercoagulable state that may result in thrombotic events that are anatomically remote from the site(s) of tumour involvement.^3^

Despite the fact that the proposed relationship between pancreatic cancer and hypercoagulability has stood the test of time, the pathological mechanism(s) responsible and the interplay among the various pathways involved are still poorly understood. Furthermore, current therapeutic options to prevent or treat thrombotic complications without increasing the risk of bleeding remain suboptimal. In the last decade, however, new insights into the biological mechanisms responsible for hypercoagulability have been reported, potentially opening the door for novel therapeutic options.^4,5^ This review summarises existing data on epidemiology and pathogenesis of thrombotic complications in pancreatic cancer. A particular emphasis has been placed on novel pathophysiological pathways.

### Epidemiology of thrombosis

#### Venous thromboembolism

The first case series describing the striking relationship between pancreatic cancer and thrombosis was published in 1938; it documented a 60% prevalence of venous thromboembolism in patients with pancreatic cancer at autopsy compared with a rate of 15–25% in other malignancies.^6^ Despite the relatively low frequency of pancreatic cancer, it was reported to account for over 17% of cancer-related thromboembolism in one retrospective analysis.^7^ Studies carried out over the past 10–15 years have reported venous thromboembolism (VTE) prevalence rates of 12–36%^8–12^ in patients with pancreatic cancer (Table 1). Lower extremity deep vein thrombosis (DVT), thrombophlebitis migrans and pulmonary embolism are the most common thromboembolic manifestations.^7^ Cancer, particularly widespread or metastatic disease, has consistently been shown to be one of the strongest risk factors for catheter-related thrombosis. A systematic review of 64 studies evaluating 29,503 adults with a central vein catheter in situ reported that the mean rates of upper extremity DVT were 4.9% overall, 6.7% in patients with cancer and 13.9% in patients admitted to critical care.^13^ The incidence of catheter-related thrombosis associated with pancreatic cancer was reported to be 1.3% in a single-centre 10-year retrospective analysis of 1915 patients with invasive exocrine pancreatic cancer at Memorial Sloan-Kettering Cancer Centre between 2000 and 2009.^8^ However, it was subsequently reported to be closer to 15% in a more recent retrospective cohort of 162 patients with pancreatic adenocarcinoma followed for an average of 15 months after diagnosis between 2004 and 2012.^14^

**Table 1:** Reported frequencies of thrombosis associated with pancreatic cancer

Study type	No. of patients	Frequency *N* (%)	Population	Reference
*Venous thrombosis*				
Retrospective	28 patients	57% (patients)	Pancreatic adenocarcinoma	^103^
	19 autopsy	47% (autopsy)		
Retrospective	380	14%	Histologically confirmed pancreatic adenocarcinoma 1949–1972	^104^
Review	94 pancreatic cancer/541 cancer-associated thrombosis	17.4% of cancer-associated thrombosis	Pancreatic malignancy	^105^
Retrospective	130	9 (6.9%)	Consecutive pancreatic adenocarcinoma	^106^
Autopsy series	154	19.4%	Consecutive autopsies 1952–1992	^107^
Retrospective	41,551	488 (11.7%)	Based on hospital discharge diagnoses 1988–1990	^108^
Retrospective	40	17.6%	Pancreatic cancer	^109^
Retrospective	438	12.1%	Included hospitalised neutropenic patients 1995–2002	^110^
Autopsy series	441	42%	Consecutive autopsies 1970–1982	^111^
Retrospective	202	Incidence rate 108.3/1000 patient-years	Consecutive patients admitted with pancreatic cancer 1990–2000	^9^
Retrospective	90	24 (26.7%)	Pancreatic adenocarcinoma	^112^
Retrospective	227	59 (26%)	Consecutive unresectable pancreatic cancer 2001–2004	^12^
Retrospective	201	58 (28.9%)	Pancreatic cancer	^113^
Retrospective	135	40 pts (29.6%)	Consecutive pancreatic adenocarcinoma patients 2006–2009	^15^
Retrospective	1915	690 (36%)	Patients receiving chemotherapy with invasive exocrine pancreatic cancer 2000–2009	^8^
Retrospective	162	28 (17.2%)	Consecutive pancreatic adenocarcinoma 2004–2012	^14^
Retrospective	475	45 (9.5%)	Consecutive pancreatic cancer receiving chemotherapy 1999–2015	^11^
*Catheter-related thrombosis*				
Retrospective	1915	9 (0.5%) 1.3% of all thrombotic events	All patients receiving chemotherapy with invasive exocrine pancreatic cancer 2000–2009	^9^
Retrospective	162	4 (2.5%) 14.3% of all thrombotic events	All patients diagnosed with pancreatic adenocarcinoma 2004–2012	^14^
*Visceral vein thrombosis*				
Retrospective	83	6 (7.2%)	Study to determine prevalence of asymptomatic thrombosis on staging CT scans in consecutive series of patients	^114^
Retrospective	135	31 patients (22.9%) 38.3% portal vein 29.8% splenic vein 27.7% mesenteric vein 2.1% hepatic vein 2.1% gonadal vein	Consecutive pancreatic adenocarcinoma patients 2006–2009	^15^
Retrospective	70	26 (38.6%)	Consecutive post-surgical patients with pancreatic exocrine cancer	^115^
Retrospective	83	26 (27.9%) splenic vein thrombosis	Consecutive patients who underwent distal pancreatectomy for adenocarcinoma 1996–2011	^116^
Prospective	136 solid cancer	12 (8%) pancreatic cancer	600 consecutive patients with visceral thrombosis 2008–2014	^16^
Retrospective	1115	132 (11.8)	Consecutive pancreatic adenocarcinoma 2005–2010 (electronic medical records) Korean population	^117^
Retrospective	1484 pancreatic ductal adenocarcinoma	95 (6.4%) 45% portal vein 17% splenic vein 26% mesenteric vein 8% gonadal vein 2% hepatic vein	Consecutive patients with pancreatic ductal adenocarcinoma and visceral thrombosis 2013–2015	^17^
*Arterial thrombosis*				
Retrospective	438	1.6%	Included hospitalised neutropenic patients 1995–2002	^110^
Retrospective	1915	30 (1.6%)	Patients receiving chemotherapy with invasive exocrine pancreatic cancer 2000–2009	^9^
Retrospective	112	SIR 2 (95% CI 1.64–2.4)	Patients with a diagnosis of cancer 1987–2008 hospitalised for coronary heart disease	^118^
Retrospective	90	SIR 2.2 (95% CI 1.8–2.7)	Patients with a diagnosis of cancer 1987–2008 hospitalised for haemorrhagic or ischaemic stroke	^119^
Retrospective	475	12 (2.5%)	Consecutive pancreatic cancer receiving chemotherapy 1999–2015	^11^
Retrospective matched-cohort	12,279	6-month cumulative incidence 5.9% MI 2.6% Ischaemic stroke 3.8%	Patients with a new diagnosis of pancreatic cancer 2002–2011	^18^

*SIR* standardised incidence ratio (calculated as the ratio of observed and expected number of coronary heart disease cases), *pts* patients, *CI* confidence intervals, *CT* computed tomography, *MI* myocardial infarction

The manifestations of systemic hypercoagulability in pancreatic cancer also include visceral vein thrombosis (Table 1). Patients who develop visceral vein thrombosis can be asymptomatic, or may present with new or increased abdominal pain, jaundice or progressive ascites. Menapace et al.^15^ reported 31 patients with visceral thrombosis among 135 patients with pancreatic adenocarcinoma (prevalence 22.9%), involving thrombosis of the portal, splenic, mesenteric, renal or gonadal veins. Interestingly, all of the visceral thrombotic events were incidentally detected on abdominal computerised tomography scanning performed for cancer staging. From a large international registry of unselected patients with visceral vein thrombosis, Ageno et al.^16^ reported that 8% of events (12/136) were associated with underlying pancreatic cancer. A retrospective cohort analysis published in 2018 evaluated patients with pancreatic adenocarcinoma with visceral thrombosis; in this series, visceral vein thrombotic events were defined as the number of major and/or minor vein branches of the splanchnic vein system discovered on diagnostic imaging. Thus, 153 events were reported among the 95 patients analysed, with a mean number of visceral thrombotic events/sites per patient of 1.6.^17^ The authors confirmed that these thrombotic events were more frequently an incidental finding on routine abdominal imaging, and that the most common location was portal vein (45%), followed by mesenteric (26%), splenic (17%) and gonadal (8%) veins.^17^ Both symptomatic and incidental vein thromboses were associated with shorter survival times in pancreatic cancer.^7,15,17^

#### Arterial thromboembolism

Arterial thromboembolic events are relatively uncommon in patients with pancreatic cancer, with an estimated incidence of 2–5%.^8^ However, a recent retrospective matched cohort of American cancer registries linked to Medicare identified 12,279 patients with pancreatic cancer and demonstrated a 6-month cumulative incidence of arterial thromboembolism of 5.9% (95% CI 5.5–6.4) compared to 2.4% (95% CI 2.1–2.7) in individuals matched by demographics and comorbidities, but without active cancer.^18^ Myocardial infarction and cerebrovascular events were most common. The 6-month cumulative incidences of myocardial infarction and ischaemic stroke were 2.6% (95% CI 2.3–2.8) and 3.8% (95% CI 3.5–4.8), respectively, in patients with pancreatic cancer, compared with 0.7% (95% CI 0.6–0.9) and 1.8% (95% CI 1.6–2.1) in control patients.^18^ As far as stroke is concerned, an observational retrospective multicentre study considered 17 patients admitted with stroke and pancreatic cancer and observed that, in 93% of cases, the cancer was metastatic.^19^ Moreover, brain imaging revealed disseminated infarction in 64% of cases, and echocardiography showed non-bacterial thrombotic endocarditis in 25% of cases. Other reported clinical presentations of arterial thromboembolism have included mesenteric and iliofemoral artery thrombosis, upper extremity ischaemia and marantic endocarditis.^7,20–22^ Simultaneous arterial and venous thromboembolism has also been described, but in the previously mentioned retrospective study of 1,915 patients, the incidence was only 0.1% (comprising 5.2% of all thrombotic complications).^8^

### Risk factors for VTE in pancreatic cancer

In cancer patients, VTE is a multifactorial event involving tumour-related factors, treatment-related factors and patient-related factors. Focusing on pancreatic cancer, the California Cancer Registry study reported the results of a multivariate analysis of potential risk factors associated with VTE within 1 year of cancer diagnosis in 6712 patients.^23^ Patients with metastatic disease at the time of diagnosis had a 3.3-fold higher risk of VTE than patients with localised disease. Notably, about 70 and 90% of patients died within 1 and 2 years of cancer diagnosis, respectively. Moreover, according to the Danish Registry, the incidence of VTE was highest in the first year following cancer diagnosis (incidence rate 56.1 (95% CI 39.9–78.8) per 1000 person-years) declining to 24.4 (7.9–75.8) per 1000 person-years during the 2nd year,^24^ possibly due to more therapeutic interventions, hospitalisations and more extensive disease during this time.

In a cohort of 202 consecutive patients with pancreatic cancer, Blom et al.^9^ reported that individuals with a tumour of the corpus/cauda (body and tail) had a 2–3-fold increased risk of VTE compared with individuals with tumours of the caput (head). Although tumours in the corpus and cauda were associated more often with distant metastases at diagnosis than tumours of the caput (70% versus 47%), after adjusting for distant metastasis, age and gender, these tumours were still associated with an increased risk of VTE (Hazard Ratio [HR] 1.6, 95% CI 0.4–5.9 and 2.5, 95% CI 0.9–7.4, respectively).^9^ Of interest, although mucin secretion by pancreatic cancer cells is one of the described prothrombotic mechanisms, no difference in adjusted VTE risk was reported in mucinous adenocarcinoma compared with non-mucinous adenocarcinoma. Patients treated with chemotherapy had a 4.8-fold increased risk (95% CI 1.1–20.8) of VTE within 3 months of discontinuing treatment, whereas radiotherapy did not increase the risk of VTE.^9^ Moreover, a 4.5-fold increased risk of VTE was observed during the 30 days postoperatively in patients who underwent surgery compared with surgical patients without cancer.^9^ Regarding individual chemotherapies, VTE occurred in 18.1% of 932 patients with various malignancies treated with a cisplatin-containing regimen at Memorial Sloan-Kettering Cancer Centre in 2008.^25^ The most common cancer associated with thrombosis was pancreatic (representing 8.5% of the cohort), and VTE occurred in 36.7% of these patients.^25^ A retrospective analysis of 227 patients with pancreatic cancer who received gemcitabine-based chemotherapy reported 15 cases of VTE (6.6%) during chemotherapy.^12^ In the study by Maraveyas et al.,^26^ 17 of 60 patients with pancreatic cancer (28.3%) receiving gemcitabine 1000 mg/m^2^ for 12 weeks developed VTE.

A recent retrospective study of 670 patients with pancreatic adenocarcinoma at the University of Texas showed that tumour location in the pancreatic corpus/cauda, previous use of antithrombotic medication, and obesity (body mass index > 30 kg/m^2^) were significant predictors for thromboembolism.^27^ Notably also, type A and AB blood groups were associated with a two-fold increased VTE risk. A better understanding of the risk factors leading to pancreatic cancer-induced thrombosis may help to identify the profile of patients who are at particularly high risk and thus most likely to benefit from prophylactic anticoagulant therapy.

### Pancreas-specific molecular risk factors for thrombosis

**Fig. 1:**
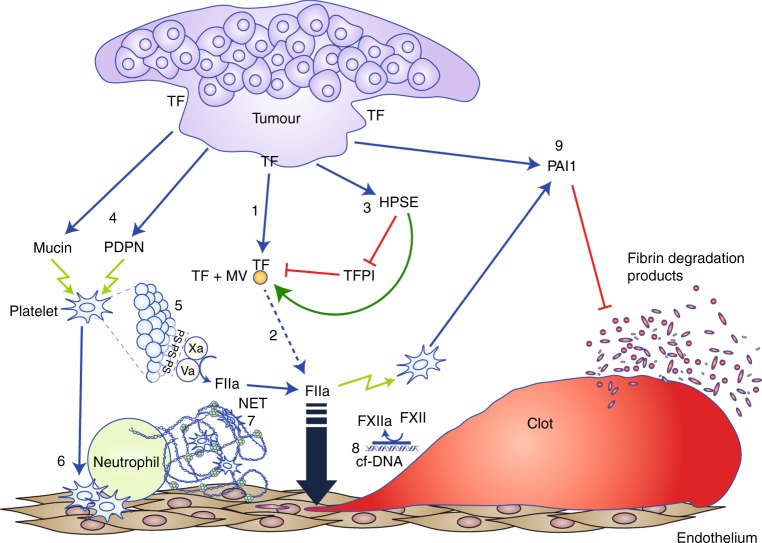
Illustration summarising the major tumour procoagulant effects in pancreatic cancer. (1) Tissue factor (TF) expression and release of TF-positive microvesicles (TF + MV) (2) TF triggers the extrinsic pathway of coagulation leading to thrombin (FIIa) generation. (3) Heparanase (HPSE) removes glycocalyces containing tissue factor pathway inhibitor (TFPI), thereby enhancing TF activity. (4) Tumour-derived mucin and podoplanin (PDPN) activate platelets, which express phosphatidylserine (PS) on their surfaces (5), facilitating prothrombinase complex assembly and thrombin generation. Activated platelets present adhesion molecules that facilitate endothelial-platelet and platelet–leukocyte interactions that contribute to generation of platelet-rich microthrombi (6). Activated neutrophils release neutrophil extracellular traps (NETs) (7) that create a matrix for blood cell and MV adhesion which promote thrombosis and impair blood flow. Cell-free DNA (cfDNA) released from tumour cells or neutrophils provides a negatively charged surface that promotes activation of factor XII (FXII) (8). FXIIa initiates the intrinsic pathway of coagulation, providing an additional source of thrombin. Plasminogen activator inhibitor 1 (PAI1)—a potent inhibitor of fibrinolysis – can be released by pancreatic tumour cells, as well as by activated platelets (9)

Because pancreatic cancer carries one of the highest rates of thrombosis, it represents an excellent model in which to study hypercoagulability in cancer (Table 2). However, while common mechanisms and risk factors might be operative across a broad spectrum of cancers, it seems likely that different forms of cancer also possess unique mechanisms that contribute to the development of thrombosis. In pancreatic cancer, significantly elevated plasma levels of fibrinogen, factor (F)VIII and D-dimer have been observed, whereas diminished levels of protein C and antithrombin III have been reported, although levels might change during tumour progression.^28,29^ More importantly, pancreatic cancer cells directly produce a number of factors, or influence a number of activities, that promote coagulation (Fig. 1).

**Table 2:** Pancreas-specific molecular risk factors for thrombosis

Molecular pathway	Mechanism	Reference
Known molecular risk factors		
High levels of procoagulants (fibrinogen, FVIII) Reduced levels of natural anticoagulants (PC, AT)	Activation of coagulation cascade	^28^
Tissue factor (TF)	Local activation of coagulation at tumour site Pro-angiogenesis (↑ VEGF, ↓ thrombospondin) Enhancement of thrombin and fibrin generation Release of tumour-TF + MVs	^7, 36, 38, 58^
Mutated or activated *KRAS2* involved in neoplastic transformation	↑ TF expression Pro-angiogenesis (↑ VEGF, ↓ thrombospondin)	^7, 43^
High tumour expression of PAI-1	Hypofibrinolysis	^39, 40^
Mucins	Platelet activation and microangiopathy	^53^
Platelets	↑ Platelet activation (PF4, P-Selectin) ↑ Procoagulant surfaces for thrombin and fibrin generation ↑ Platelet-leukocyte interactions ↑ Platelet adhesion to endothelium ↓ Local fibrinolysis Induction of NETs formation	^4, 41, 44, 49, 50, 53– 55^
Cytokine release (IL-1, TNF-α, VEGF)	Proinflammatory mechanisms ↑ TF production by vascular endothelial cells Downregulation of TM expression ↑ PAI-1 synthesis ↑ Endothelial expression of adhesion molecules	^41, 42^
Novel molecular risk factors		
TF + MVs	↑ Procoagulant surfaces for thrombin and fibrin generation Trigger coagulation via the extrinsic pathway	^33, 56– 60^
Heparanase (HPSE)	↑ TF expression ↓ TFPI on endothelial and tumour cells surface	^62, 63^
NET-hypercitrullinated histone H3	Capture platelets and MVs for clot stabilisation TFPI inactivation by elastase and cathepsin G ↑ Platelet adhesion and thrombus formation under shear stress	^4, 67, 68^
Cell-free DNA	FXII-dependent procoagulant activity	^70, 74^
Contact activation (various proposed pathways)	Trigger the initiation of coagulation via the intrinsic pathway	^70^
Podoplanin (PDPN)	↑ Platelet aggregation Release of PDPN-bearing MVs	^81, 82^

*F* factor, *PC* protein C, *AT* antithrombin, *MVs* microvesiscles, *VEGF* vascular endothelial growth factor, *uPA* urokinase plasminogen activator, *PAI-1* plasminogen activator inhibitor 1, *TM* thrombomodulin, *PF4* platelet factor 4, *NETs* neutrophil extracellular traps, *TF* *+* *MVs* tissue factor-bearing MVs, *TFPI* tissue factor pathway inhibitor

#### Tissue factor

**Fig. 2:**
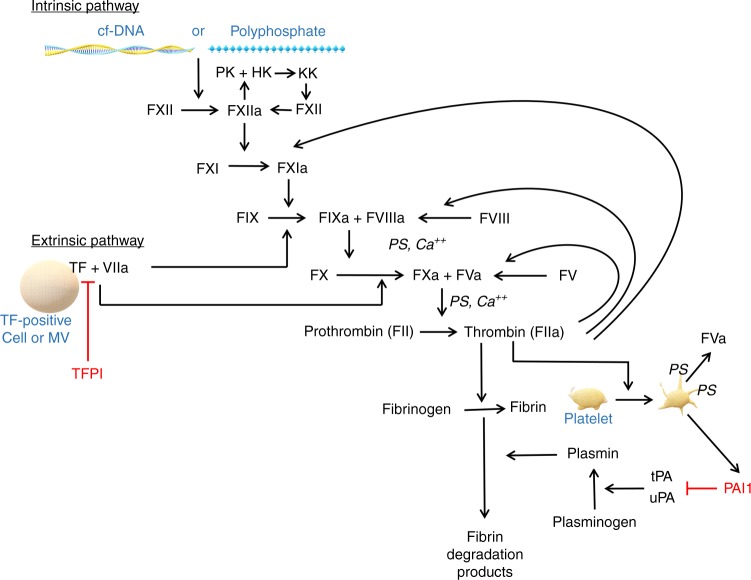
Mechanisms of haemostasis and fibrinolysis. Activators of both the intrinsic and extrinsic pathways are shown. Abbreviations: calcium ion (Ca^++^), cell-free deoxyribonucleic acid (cfDNA), high molecular weight kininogen (HK), prekallikrein (PK), kallikrein (KK), microvesicle (MV), phosphatidylserine-containing phospholipid (PS), tissue factor (TF), tissue factor pathway inhibitor (TFPI), tissue type plasminogen activator (tPA), urokinase type plasminogen activator (uPA), plasminogen activator inhibitor 1 (PAI1)

Of prime importance in pancreatic cancer-mediated hypercoagulability is tissue factor (TF), a transmembrane receptor that initiates the extrinsic pathway of coagulation. TF is not expressed on the membrane of quiescent endothelial cells, but is present in vascular smooth muscle cells.^7^ Although breast tumour-associated endothelial cells have been observed to express TF,^30^ this finding was not independently confirmed^31^ and, to our knowledge, has not been unequivocally established in the case of endothelial cells associated with pancreatic tumours. However, vascular endothelial cell disruption leads to exposure of subendothelial TF to blood. Exposed TF can thus bind to factor VII (FVII), which then becomes activated (FVIIa). The TF/FVIIa complex converts factor X into factor Xa, and FXa in turn activates prothrombin leading to formation of thrombin (factor IIa) (Fig. 2). Importantly, TF is expressed in exocrine pancreatic cells upon malignant transformation and is also expressed on inflammatory and stromal cells within the tumour microenvironment.^32^ In these locations, TF is believed to be a key factor in both thrombosis and metastatic spread.^33,34^ For example, a study of 41 pancreatic cancers showed a higher prevalence of VTE in patients with higher levels of tumour TF expression (26.3%) compared with those with low tumour TF expression (4.5%).^33^

Engagement of TF by FVIIa not only initiates the coagulation cascade but also activates protease-activated receptor 2 (PAR2) signalling after β_1_-integrin ligation, thereby contributing to angiogenesis, tumour cell proliferation and migration.^35^ Wojtukiewicz et al.^36^ showed substantial in situ TF expression in pancreatic carcinoma, along with other procoagulants, including prothrombin and fibrinogen. By contrast, staining for the anticoagulant and antiangiogenic protein tissue factor pathway inhibitor (TFPI) and for plasminogen activators was minimal. Furthermore, TF contributes to tumour-directed angiogenesis by upregulating the expression of vascular endothelial growth factor (VEGF) and downregulating the expression of the angiogenesis inhibitor, thrombospondin. The expression of TF has been shown to correlate with the histological grade of the tumour: specifically, immunohistochemical analysis showed that 77% of poorly differentiated pancreatic tumours expressed TF compared with 20% of well-differentiated tumours, indicating an association between the presence of TF with more aggressive histological subtypes.^34^ Moreover, an investigation into the prognostic significance of TF expression in pancreatic ductal carcinoma showed that increased TF expression correlated positively with the size of the primary tumour, lymph node metastasis, distant lymphatic metastasis, advanced TNM stage and higher tumour grade; more importantly, high TF expression was an independent negative predictor of survival.^37^

A major result of TF pathway activation in pancreatic cancer is accelerated thrombin generation, which is outlined in Fig. 2. Functional thrombin receptors have been identified in human pancreatic cancer cells but not in healthy pancreatic tissue.^38^ Aside from its procoagulant roles, thrombin enhances the adhesion of pancreatic adenocarcinoma cells to extracellular matrix proteins and to endothelial cells, suggesting that it is important for pancreatic tumour growth and invasion.^7^

#### Fibrinolysis

In addition to promoting the formation of fibrin clots, cancer can also lead to inhibition of fibrinolysis, the process by which blood clots are broken down. Human pancreatic tumours and cell lines have been shown to express plasminogen activator inhibitor type 1 (PAI-1), a key inhibitor of fibrinolysis,^39^ and a study of patients with pancreatic cancer has indicated that elevated plasma levels of PAI-1 antigen and its associated activity might predispose patients to VTE.^40^

#### Cytokines

Malignant pancreatic cells also secrete a variety of inflammatory cytokines, including interleukin (IL)-1, tumour necrosis factor (TNF)-α and VEGF.^41^ In vitro, these cytokines can induce TF production by vascular endothelial cells, downregulate the endothelial expression of thrombomodulin (the normal function of which is to control thrombin generation), promote PAI-1 synthesis and increase the expression of heterocellular adhesion molecules in endothelial cells.^42^ All these mechanisms probably contribute to hypercoagulability because they render the vascular endothelium more liable to promote thrombosis.

#### Neoplastic transformation and coagulopathy

It has been suggested that the products of genes involved in neoplastic transformation might act as mediators of cancer coagulopathy. Such candidates include KRAS and c-MET, and p53.^10^ Mutated or activated KRAS2 (found in 95% of pancreatic cancers) has been shown in mouse models to increase thrombosis by increasing the expression of tissue factor.^43^

#### Platelet activation

Platelet aggregation induced by tumour cells is a key contributor to the prothrombotic state in pancreatic cancer. Pancreatic cancer cell lines induce platelet aggregation in vitro via a thrombin-dependent mechanism.^44^ Mucin-producing pancreatic carcinomas have also been associated with thrombosis in a thrombin-independent manner, mediated by platelet activation and a resulting microangiopathy. Mucins are high molecular weight glycoproteins that have O-glycosylated tandem repeat regions rich in proline, threonine and serine residues. Some pancreatic cancers express aberrantly glycosylated structures on their cell surface and shed large quantities of mucins into the circulation.^45^ Wahrenbrock and colleagues^46^ showed that injection of highly purified mucin into mice resulted in the rapid generation of platelet-rich microthrombi, and that thrombosis was markedly diminished in mice deficient in the mucin receptors, P-selectin or L-selectin. In the absence of a method to quantify mucins in blood, carbohydrate antigen CA19-9, a widely used tumour marker for pancreatic cancer, could serve as a surrogate marker, as it binds to apomucins, including MUC1, MUC5AC and MUC16.^47^ Woei-A-Jin et al.^48^ reported that the plasma levels of CA19-9 correlated with increased stage, shortened survival and severity of VTE in 79 patients with pancreatic cancer.

Numerous studies have shown that plasma levels of biomarkers of platelet activation (i.e. soluble P-selectin, soluble CD40 ligand, thrombospondin-1 and ß-thromboglobulin) were significantly higher in cancer patients than in healthy controls, strongly suggesting that platelet activation is relatively common during cancer progression.^49^ The platelet activation marker platelet factor 4 (PF4) has also been proposed as a prognostic biomarker in pancreatic cancer, as high PF4 serum levels (>11 kU/ml) have been associated with an increased risk for VTE and poor survival.^50^ Moreover, cancer-cell capability to alter platelet RNA and protein profiles to control systemic and local responses to tumour growth has been described in pancreatic cancer, leading to the notion of “tumour-educated platelets”.^51^ Interestingly, Best et al.^52^ were able to distinguish cancer patients (including 35 pancreatic) from healthy individuals by sequencing platelet mRNA with 96% accuracy. Several mechanisms have been proposed for platelet involvement in VTE.^49^ First, activated platelets exhibit a procoagulant surface by exposing negatively charged phospholipids that can induce thrombin generation, ultimately leading to fibrin formation. Second, platelet–leukocyte interactions appear to be crucial for the generation of platelet-rich microthrombi in adenocarcinomas, as already mentioned.^53^ Third, platelet adhesion to the endothelium was demonstrated to be a critical step for thrombosis in mouse models.^54^ Fourth, activated platelets are known to release inhibitors of fibrinolysis (such as PAI-1), creating a local hypofibrinolytic state at the site of the clot.^55^ Fifth, platelet interaction with neutrophils have been shown to induce the formation of neutrophil extracellular traps (see below).

### Novel molecular risk factors for cancer-associated thrombosis in pancreatic cancer

Additional molecules/factors have been identified as potential mediators of cancer-associated hypercoagulability over the past decade (Table 2). These molecules/factors might also have the potential to be used as clinically informative biomarkers in the prediction of thrombotic complications.

#### Microvesicles

As the presence of procoagulant TF on tumour cells does not explain why thrombosis typically occurs at sites distant from the primary tumour, it has been hypothesised that TF borne by circulating microvesicles might be a contributing factor.^56^ Indeed, cultured TF-expressing cancer cells can release TF-bearing microvesicles into the culture supernatant, and TF-expressing tumours release TF-positive microvesicles into the blood. TF on the surface of microvesicles is also available to bind plasma FVII(a), and therefore can trigger coagulation and promote thrombosis at sites remote from the tumour. It has been shown in patients with pancreatic cancer that TF-positive microvesicles co-express the epithelial tumour antigen MUC1 and that surgical pancreatectomy dramatically reduces the circulating level of these microvesicles.^57^ Direct evaluation of the procoagulant properties of these microvesicles has been demonstrated in mouse models. Injection of TF-positive microvesicles isolated from a human pancreatic cell line increased venous thrombosis when injected into mice, while inhibition of TF prevented this effect.^58^ Several studies have described the presence of TF-positive microvesicles in the circulation of patients with solid cancers, with the highest levels of microvesicle-TF activity reported in patients with pancreatic cancer. One study on 30 patients with pancreatic adenocarcinoma measured circulating TF-positive microvesicles both by flow-cytometry and by a functional procoagulant assay, and showed a slight reduction in the number of microvesicles with a significant reduction of microvesicle-TF procoagulant activity.^59^ Interestingly, higher numbers of circulating TF-positive microvesicles or microvesicle-TF activity correlated with TF expression and microvessel density on tumour tissues. However, not all studies have shown a clear association between circulating microvesicle-TF activity and VTE occurrence in pancreatic cancer.^56^ In addition, the intensity of TF expression by adenocarcinoma cells does not necessarily correlate with plasma microvesicle-TF activity.^48^ Moreover, a study involving three pancreatic cancer patients showed that 50% of TF-positive microvesicles were MUC1-negative,^60^ indicating a source of TF-positive microvesicles separate from tumour cells. TF-bearing microvesicles can also be released from activated macrophages or other sub-endothelial sources. In fact, large clusters of tumour-invading macrophages that stained strongly for TF have been observed in pancreatic tissue specimens from patients with high plasma microvesicle-TF activity. Woei-A-Jin and colleagues^48^ hypothesised that the pro-inflammatory state in advanced pancreatic cancer induces the activation of monocytes and macrophages to express large quantities of TF, and that both TF-positive tumour cells and activated TF-positive macrophages within the tumour environment are the source of circulating TF-positive microvesicles.

#### Heparanase

Among other tumour cell procoagulant molecules, the enzyme heparanase (HPSE) is gaining increasing attention. HPSE is a multitasking endo-β-D-glucuronidase that is capable of cleaving heparan sulphate side chains from heparan sulphate proteoglycans on cell surfaces and in the extracellular matrix,^61^ thereby participating in extracellular matrix degradation and remodelling. This activity is strongly implicated in tumour metastasis and angiogenesis, but HPSE might also affect cancer and coagulation in a non-enzymatic manner. In cancer, enzymatically-inactive HPSE was showed to facilitate adhesion and migration of endothelial cells, induce VEGF, and facilitate the formation of lymphatic vessels.^61^ As for HPSE non-enzymatic procoagulant activity, Nadir and Brenner^61^ showed that HPSE upregulated the expression of TF and interacted with TFPI on the cell surface of endothelial and tumour cells, leading to dissociation of TFPI from these cellular surfaces with a net increase in cell surface procoagulant activity. Additionally, HPSE directly enhances TF activity, leading to increased generation of factor Xa.^61^

In a 2018 study, HPSE mRNA in the peripheral blood mononuclear cell fraction and HPSE activity in plasma and urine were detected in 31 patients with pancreatic cancer. Both HPSE expression and activity decreased significantly in 17 patients following tumour resection, but increased markedly in six patients, coincident with recurrence or metastasis. HPSE mRNA and activity also decreased in patients who received chemotherapy.^62^ Thus, HPSE levels might correlate with prognosis in patients with pancreatic cancer. Moreover, overexpression of HPSE is associated with increased infiltration of tumour-associated macrophages in both mouse and human pancreatic ductal adenocarcinoma, and with a pro-inflammatory and pro-tumour profile of these macrophages.^63^ It is possible that HPSE overexpression in pancreatic tumour tissue might alter the macrophage profile, leading to an enhanced generation of TF-positive microvesicles and contributing to the prothrombotic state. However, no study has yet reported an association between HPSE and thrombotic complications in pancreatic cancer.

#### Neutrophil extracellular traps

Several studies have reported that leukocytosis that accompanies cancer is associated with an increased risk of VTE.^64^ Apart from the capacity of monocytes/macrophages to express procoagulant TF and produce TF-positive microvesicles, highly activated neutrophils might enhance thrombosis by generating neutrophil extracellular traps (NETs).^4^ NETs are composed of extracellular DNA fibres, histones and granular proteins that have been shown to play a role in innate immunity by enhancing the proteolysis and killing of bacteria. The process of NETosis requires the enzyme, peptidylarginine deiminase 4 (PAD4), which converts arginine residues into citrulline on histones, thereby facilitating the unwinding of DNA and its ensuing elimination from neutrophils. Therefore, a proposed biomarker of in vivo NETosis is circulating hypercitrullinated histone H3.^65^ One study demonstrated that citrullinated histone H3 is present in both human and murine pancreatic tumours, suggesting that infiltrating neutrophils release NETs into the tumour microenvironment.^66^ The process of autophagy, in which damaged organelles and proteins are degraded and recycled, is also critical for NET formation.^66^ Autophagy is an important regulator of cancer cell survival in pancreatic cancer, allowing cells to survive the hypoxic, nutrient-deprived tumour microenvironment.

NETs have been shown to contribute to VTE in animal models.^64^ One of their major procoagulant roles may be to capture platelets and microvesicles that propagate clot formation.^67^ In addition, NETs indirectly promote TF activity by binding elastase and cathepsin G, which can inactivate TFPI.^68^ Most inducers of NET formation studied thus far drive a NET-releasing mechanism that requires prolonged neutrophil stimulation (and ultimate cell death) in a process that is dependent on the generation of reactive oxygen species (ROS). Interestingly, however, pancreatic cancer cells are able to stimulate rapid and ROS-independent NETosis.^4^ Although anucleate platelets do not produce NETs, when stimulated by bacteria, viruses, or traditional agonists, they may bind to and activate neutrophils to release NETs. This interaction can be beneficial to protect the host against pathogens but, if uncontrolled, can cause tissue and organ damage.^69^ A trend towards increased NET release promoted by platelets pre-stimulated by pancreatic cancer cells has been reported.^4^ Of interest, neutrophils isolated from two distinct animal models of pancreatic ductal adenocarcinoma demonstrated an increased propensity to form NETs following stimulation with platelet-activating factor.^66^ Furthermore, NETs promote platelet adhesion and thrombus formation under venous shear stress ex vivo.^4^ These studies demonstrate that NET formation is upregulated in pancreatic cancer, and that, in turn, the so-formed NETs act as a platform for additional platelet adhesion and thrombus formation.

#### Contact system activation

The intrinsic contact coagulation pathway is initiated by negatively charged surfaces that promote conversion of zymogen FXII into the activated serine protease FXIIa. One of the end-results of FXIIa generation is FXI cleavage to generate activated FXI (FXIa). FXIa initiates a series of Ca^2+^-dependent proteolytic events that lead to thrombin generation, and production of a fibrin clot^70^ (Fig. 2). Both neutrophils and tumour cells can release nucleic acids, such as DNA, mRNA and microRNA, and cancer patients have elevated levels of cell-free DNA (cfDNA) in their blood.^71^ Mutated cfDNA with the pancreatic-tumour-specific *KRAS* mutation has been proposed as a promising diagnostic and prognostic biomarker in pancreatic adenocarcinoma.^72^ Furthermore, cancer chemotherapy is also associated with increased plasma levels of cfDNA.^65,73^ Multiple groups have reported FXII-dependent procoagulant activity of purified DNA in vitro.^70,74^ Specifically, Swystun et al.^73^ showed that cfDNA purified from epirubicin-treated whole blood ex vivo significantly elevated thrombin generation in a dose-dependent manner by a mechanism involving activation of the intrinsic contact activation pathway. No study has so far addressed the role of cfDNA in pancreatic-cancer-associated thrombosis.

Early evidence of contact system activation in cancer was presented in 1990 in a case-control study, which evaluated 69 patients with gastrointestinal cancer (12 with gastric cancer, 15 with pancreatic cancer and 42 with colon cancer), 33 of whom had liver metastases, and 118 healthy controls.^75^ The authors showed a pattern of reduced contact factor levels with markedly elevated inhibitor levels, which is compatible with systemic contact activation.^75^ More recently, Pan et al.^76^ confirmed the presence of contact system activation in several solid cancers (10 lung, 11 colon, nine breast, three pancreatic and one renal). In particular, all samples from patients with pancreatic cancer showed undetectable levels of high molecular weight kininogen (HK), whereas only one of the colon cancer plasmas had undetectable HK, which occurred in a patient with stage IV disease. Rousseau et al.^77^ measured thrombin generation in normal plasma in the presence of pancreatic adenocarcinoma cells (BXPC3) or breast cancer cells (MCF7). The effect of plasma depleted of individual clotting factors or the addition of an inhibitor to TF (monoclonal antibody) or FXIIa (corn trypsin inhibitor (CTI)) on thrombin generation was tested. Interestingly, anti-TF had more inhibitory activity on thrombin generation triggered by BXPC3 cells while CTI had more effect on thrombin generation triggered by MCF7 cells. Using FXI- or FXII-depleted plasma, thrombin generation was more decreased with MCF7 than with BXPC3 cells. The authors concluded that thrombin generation by BXPC3 cells was mainly driven by the TF pathway,^77^ but the same was not true of MCF7 cells. Although several observations documenting contact system activation in various cancers have been published, the responsible molecular mechanism is poorly understood. However, several candidates that are found in the circulation of patients with cancer, including cfDNA, microvesicles and polyphosphates, are known to activate the contact system in vitro.^70^

#### Podoplanin

Podoplanin (PDPN), a 38-kDa type I transmembrane glycoprotein, is normally expressed in kidney podocytes, alveolar type I cells, osteocytes, basal keratinocytes, mesothelial cells and lymphatic endothelial cells,^78^ but its expression can be upregulated on the surface of different cancer cells and cancer-associated fibroblasts by tumour promoters including RAS, Src and the phorbol ester, 12-*O*-tetradecanoylphorbol-13 acetate (TPA). Src activity is associated with many types of human cancer, including tumours of the colon, breast, pancreas, brain and skin.^78^ Hirayama et al.^79^ evaluated the PDPN expression in tumour samples obtained from 95 patients with invasive ductal carcinoma of the pancreas and found that PDPN expression in cancer-related fibrotic tissues was associated with a poor prognosis, especially in patients with large tumours or lymph node metastases. The proposed prothrombotic mechanism of PDPN is activation of platelets via the C-type lectin-like receptor 2 (CLEC-2). In fact, evidence suggests that PDPN and CLEC-2 play a mechanistic role in the process of thrombus formation both in an inferior vena cava stenosis mouse model of DVT, where both inhibition of PDPN as well as CLEC-2 deficiency resulted in significantly reduced extension of thrombosis, and in an in vivo mouse model of infection-driven thrombosis.^80^ In patients with brain tumours, PDPN overexpression is strongly correlated with the presence of intratumoural thrombotic vessels, hypercoagulability and increased VTE risk. Moreover, in vitro experiments demonstrated that platelet aggregation induced by human glioblastoma cells was highly dependent on PDPN.^81^ In brain tumours, it has been suggested that PDPN might be released from the primary tumour into the bloodstream, where it can potentially promote VTE development. Interestingly, PDPN-bearing microvesicles derived from tumour cells were observed in the circulation of patients with pancreatic and colorectal cancer, implying a possible prothrombotic role for PDPN in pancreatic cancer, also.^82^

### Novel therapeutic scenarios in cancer-associated thrombosis

The treatment of cancer-associated thrombosis is challenging, because recurrent thrombosis and bleeding occur simultaneously reasonably often.^29,42,83^ In the future, however, it is hoped that new approaches will be able to reduce thrombotic recurrence without impairing haemostasis (Table 3).

**Table 3:** Novel potential therapeutic strategies in cancer-associated thrombosis

Target molecule	Therapeutic strategy	Evidence	Reference
Tissue factor (TF)	Anti-human TF mAb (HTF-1)	↓ Clot size in mice bearing human orthotopic pancreatic tumour mice but did not affect clot size in healthy mice	^84^
	Anti- phosphatidylethanolamine (duramycin)	1. ↓ Thrombus weight and incidence 2. Systemic administration had no significant effect on blood coagulation and bleeding time	^5^
Platelets	Clopidogrel	1. ↓ Binding of tumoural MVs to the site of thrombosis in murine orthotopic pancreatic cancer model 2. ↓ Thrombosis induced by TF + MVs in murine pancreatic cancer model	^87, 88^
	Blocking rat anti-mouse P-Selectin mAb	↓ Thrombosis in a mesenteric ferric chloride model in mice with pancreatic cancer	^89^
Heparanase	Peptide derived from TFPI-2 first Kunitz domain	Inhibition of coagulation activation in cancer-bearing mice No bleeding tendency	^90^
NETs	Recombinant human DNase I	Abolished thrombus formation in a murine breast cancer model	^91^
	Autophagy inhibition by chloroquine	1. ↓ NET formation by neutrophils and in the pancreatic tumour microenvironment 2. ↓ Levels of citrullinated histone H3	^66^
Factor XIIa	Corn trypsin inhibitor (CTI)	↓ Thrombin generation by MVs isolated from pancreatic cancer patients	^97^
Podoplanin (PDPN)	Neutralising human anti-PDPN mAb (NZ-1)	↓ PDPN-dependent platelet aggregation	^98^
	Direct binding activity to CLEC-2 (2CP)	Chemical inhibition of PDPN-induced platelet aggregation No defects in physiological platelet function	^100^
	Anti-CLEC-2 mAb 2A2B10	Abolished thrombus formation of PDPN-positive melanoma bearing- mice No significant bleeding tendency	^101^
PAI-1	Oral inhibitor of active PAI-1 (PAI-039)	Blocked bevacizumab-induced thrombosis in human lung adenocarcinoma-bearing mice	^102^

*mAb* monoclonal antibody, *TF* tissue factor, *MVs* microvesicles, *TFPI-2 TF* pathway inhibitor 2, *NETs* neutrophil extracellular traps, *CLEC-2* C-type lectin-like receptor-2, *PAI-1* plasminogen activator inhibitor 1

#### Targeting TF, microvesicles and platelets

Hisada et al.^84^ demonstrated that mice with human orthotopic BXPC-3 pancreatic adenocarcinoma tumours had significantly larger clots than control mice in a model of venous thrombosis; furthermore, clots from tumour-bearing mice contained human TF, suggesting the incorporation of circulating tumour-derived microvesicles. Importantly, the administration of an anti-human TF monoclonal antibody reduced clot size in tumour-bearing mice, demonstrating that inhibition of TF could be a novel strategy to reduce VTE in patients with pancreatic cancer.^84^ It is worth mentioning that, as TF expression is an important determinant of pancreatic cancer growth and progression, the use of therapeutic anti-TF has been described to significantly reduce tumour growth in a mouse model of pancreatic cancer, leading to the possibility of concomitant treatment of cancer and cancer-related thrombosis.^85^ A study by Stark et al.^5^ confirmed the role of pancreatic-tumour-derived microvesicles in amplifying thrombosis in a murine model of flow restriction of the vena cava. Specifically, the authors demonstrated a synergistic activation of coagulation by TF expressed by pancreatic-tumour-derived microvesicles and host TF. Interestingly, targeting phosphatidylethanolamine (a phospholipid expressed on the surface of microvesicles) using duramycin selectively prevented microvesicle-associated DVT without inducing bleeding, presumably by impairing TF/FVIIa-dependent FXa generation on the microvesicle surface.^5^ In contrast, the direct thrombin inhibitor dabigatran was less effective in preventing tumour microvesicle-associated DVT. In a clinical setting, a randomised phase 2 study (the Micro-TEC study) evaluated the role of enoxaparin thromboprophylaxis in patients with high circulating levels of TF-positive microvesicles. Of the 66 cancers, 30 were pancreatic.^86^ There was a trend towards reduced VTE at 2 months in patients with higher TF-positive microvesicle numbers randomised to enoxaparin compared with those randomised to observation (5.6% versus 27.3%, *P* = 0.06).

A role for antiplatelet agents in preventing thrombosis associated with cancer has also been described in mouse models. One study, using a syngeneic orthotopic model of pancreatic cancer, found that clopidogrel reduced the binding of tumour-derived microvesicles to thrombi,^87^ while another group reported that enhancement of thrombosis in mice by injection of exogenous TF-positive microvesicles was reduced by clopidogrel.^88^ Moreover, it has been shown that inhibition of P-selectin reduced thrombosis in the mesenteric ferric chloride model in mice with pancreatic tumours, with no effect on thrombosis in non-tumour-bearing mice,^89^ indicating that activated platelets and endothelial cells expressing P-selectin as well as microvesicle-borne P-selectin glycoprotein ligand 1 are involved in thrombus formation in this model. However, there are no clinical studies on the use of antiplatelet therapy to prevent VTE in pancreatic cancer.

#### Targeting HPSE and NETs

A further possible antithrombotic strategy concerns inhibition of the procoagulant activity of HPSE. Peptides derived from the solvent-accessible surface of TFPI-2’s first Kunitz domain inhibited the interaction between TF and HPSE. In vivo, these newly identified peptides attenuated activation of the coagulation system and reduced sepsis severity without predisposing to significant bleeding in a mouse model.^90^

Systemic administration of DNAse confers protection against experimental models of cancer-associated thrombosis.^91^ Notably, DNAse I does not impair haemostasis. Convincing evidence supporting the contribution of NETs to human thrombosis and clinical experience with systemic administration of DNAse I in humans are currently lacking. However, treatment of murine models and pancreatic cancer patients with the autophagy inhibitor chloroquine reduced NET formation in circulating neutrophils and decreased NET production in the tumour microenvironment.^66^ Furthermore, patients who had a CA19-9 response to treatment with autophagy inhibition and chemotherapy had lower levels of citrullinated histone H3 staining, suggesting that a greater response to treatment resulted in greater inhibition of NETs in the tumour microenvironment. More specifically, one of the detrimental effects of NETs is the promotion of inflammation in the tumour microenvironment, inducing changes in the phenotype of infiltrating immune cells or stromal fibroblasts that subsequently promote tumour growth.^66^ Thus, autophagy inhibition has the potential to reduce tumour growth by reducing inflammation in the microenvironment. Current data suggest that the effects of autophagy inhibition may extend beyond the tumour microenvironment; in fact, autophagy inhibition has also been showed to chemosensitise pancreatic cancer cell lines to doxorubicin.^92^ Autophagy inhibition with chloroquine or hydroxychloroquine in cancer has been evaluated in several phase 2 and 3 clinical trials. A recent meta-analysis, including seven clinical trials, three of which were phase 2 trials in pancreatic adenocarcinoma, concluded that autophagy-inhibitor-based therapy was associated with improved progression-free survival (RR 1.72, 95%CI 1.05–2.82) and overall survival (RR 1.39, 95%CI 1.11–1.75) than standard chemotherapy.^93^ Additionally, an inhibitor of PAD4, Cl-amidine, has been shown to have beneficial effects in murine models of breast and colon cancer,^66,94,95^ suggesting that the role of PAD4 inhibitors in the treatment of pancreatic cancer should also be explored.

#### Targeting the intrinsic pathway

TF is very efficient at initiating coagulation in pancreatic cancer-associated thrombosis. However, thrombus formation also relies on the intrinsic pathway for subsequent thrombin generation. FXI can be activated by either FXIIa or by thrombin. Consequently, FXI or FXIa are potentially attractive anti-thrombotic targets. Inhibition of the contact system might protect against thrombosis without increasing the risk of bleeding, as previously shown by genetic or pharmacologic inhibition of FXIIa in animal models.^96^ Several classes of contact system inhibitors are currently undergoing development as thromboprotective and/or anti-inflammatory agents, and have recently been reviewed.^70^ Most of these agents, however, have demonstrated the ability to inhibit the contact system in vitro or in experimental models of thrombosis in animals without cancer. Hellum et al.^97^ showed that in contrast to microvesicles from healthy controls, microvesicles from patients with pancreatic cancer generated lower levels of thrombin in the presence of corn trypsin inhibitor, possibly indicative of microvesicle-mediated promotion of the contact system.

#### Targeting PDPN

Kato et al.^98^ developed a cancer-specific neutralising monoclonal antibody against human PDPN, which blocked the association between PDPN and CLEC-2. This agent demonstrated the ability to inhibit PDPN-dependent platelet aggregation and PDPN-induced cancer metastasis.^98^ Although this cancer-specific antibody shows promise as a molecular targeting therapy, PDPN is expressed only on the stromal fibroblasts that surround tumours of the pancreas. However, Suzuki-Inoue et al.^99^ reported that PDPN that is expressed in cancer cells might be involved in migration, invasion and metastasis by promoting platelet aggregation. Thus, PDPN expressed in stromal fibroblasts around pancreatic tumours might be involved in cancer progression and increased thrombotic risk, via mechanisms mediated by multiple growth factors derived from activated platelets. Although there are numerous anti-PDPN monoclonal antibodies, tools targeting CLEC-2 are much more limited. Interestingly, a small molecule compound, 2CP, has been reported as a chemical inhibitor of PDPN-induced platelet aggregation.^100^ 2CP binds directly to CLEC-2 and shows therapeutic efficacy in combination with cisplatin without affecting physiological platelet function in a mouse metastasis model. In another recent report, immunological depletion of CLEC-2 in mice using the anti-CLEC-2 mAb 2A2B10 suppressed haematogenous metastasis and thrombus formation of the PDPN-positive mouse melanoma cell B16F10, without inducing any significant bleeding.^101^ Further investigations are needed to evaluate therapies targeting PDPN in mitigating the thrombotic risk in pancreatic cancer.

#### Targeting hypofibrinolysis

The potential therapeutic role of targeting hypofibrinolysis has been evaluated in a mouse model of human lung adenocarcinoma. Administering the anti-VEGF agent bevacizumab in this model promoted thrombosis by simultaneously increasing PAI-1 expression in tumours and in plasma, an effect that was reduced by a PAI-1 inhibitor.^102^ Further studies are needed to determine the role of hypofibrinolysis mediated by PAI-1 in pancreatic-cancer-associated thrombosis.

## Conclusions

Pancreatic cancer remains one of the most prothrombotic neoplasms, with an incidence of thrombotic complications of up to 36%. Thus, it is an excellent model in which to study cancer-associated hypercoagulability. Pancreatic cancer, however, is characterised by the peculiarity of a high level of expression of TF in tumour tissue and the release of tumour-derived microvesicles that might promote distal thrombosis by activating both the extrinsic and intrinsic pathways, as well as by promoting platelet adhesion and activation, and the release of NETs from leukocytes. Furthermore, novel coagulation markers and pathways might contribute to these processes, including HPSE, PDPN and the fibrinolytic system. Importantly, pancreatic cancer has to be considered a dynamic milieu of cellular and acellular elements including fibroinflammatory stroma, extracellular matrix and infiltrating immune cells, in addition to the cancer cell population. Thus, we postulate that pancreatic cancer-induced hypercoagulability is the result of procoagulant properties of cancer cells themselves together with the procoagulant properties of elements present in the microenvironment. Recent data derived from preclinical mouse models seem to suggest novel therapeutic possibilities, with the goal of treating individual patients with specific cancer types at particular stages of the disease. This is an exciting era for cancer-associated thrombosis with the discovery of novel prothrombotic and proinflammatory mechanisms, and the potential for new pharmacological targets that will allow prevention and treatment of thrombosis without affecting haemostasis.

## Data Availability

Data sharing is not applicable to this article as no datasets were generated or analysed during the current study.
